# Plasma Rich in Growth Factors Compared to Xenogenic Bone Graft in Treatment of Periodontal Intra-Osseous Defects—A Prospective, Comparative Clinical Study

**DOI:** 10.3390/jfb15110336

**Published:** 2024-11-09

**Authors:** Sourav Panda, Sital Panda, Abhaya Chandra Das, Natalia Lewkowicz, Barbara Lapinska, Margherita Tumedei, Funda Goker, Niccolò Cenzato, Massimo Del Fabbro

**Affiliations:** 1 Department of Periodontics, Institute of Dental Sciences, Siksha ‘O’ Anusandhan University, Bhubaneswar 751002, Odisha, India; abhayadas@soa.ac.in; 2 Research Associate, Institute of Dental Sciences, Siksha ‘O’ Anusandhan University, Bhubaneswar 751002, Odisha, India; drsitalpanda@gmail.com; 3 Department of Periodontology and Oral Diseases, Medical University of Lodz, 251 Pomorska St., 92-213 Lodz, Poland; natalia.lewkowicz@umed.lodz.pl; 4 Department of General Dentistry, Medical University of Lodz, 251 Pomorska St., 92-213 Lodz, Poland; barbara.lapinska@umed.lodz.pl; 5 Department of Biomedical, Surgical and Dental Sciences, Università degli Studi di Milano, 20122 Milan, Italy; margherita.tumedei@unimi.it (M.T.); funda.goker@unimi.it (F.G.); niccolo.cenzato@unimi.it (N.C.); 6 Fondazione IRCCS Ca’ Granda Ospedale Maggiore Policlinico, 20122 Milan, Italy

**Keywords:** intra-osseous defects, periodontal regeneration, plasma rich in growth factors, xenograft

## Abstract

Background: Periodontal intra-bony defects are challenging conditions in dental practice, often requiring regenerative approaches for successful treatment. This clinical study aimed to compare the effectiveness of plasma rich in growth factors (PRGF) versus xenogenic bone graft (BXG) in addressing intra-bony defects. Methods: Forty patients aged between 30 and 50 years presenting with generalized periodontitis were included. The study assessed various parameters, including relative attachment level (RAL); probing pocket depth (PPD); gingival marginal level (GML); intra-bony defect depth (IBDD) at baseline, 3, and 6 months; and level of pain, post-operative bleeding, and swelling, as patient-reported outcomes during the first seven days post operation. Results: The results revealed that both PRGF and BXG treatments led to significant reductions in IBDD over the 6-month study period. PRGF demonstrated significant advantages in GML enhancement and post-operative pain management during the initial post-treatment days. However, BXG showed a significantly greater reduction in IBDD compared to PRGF. Post-operative bleeding and swelling levels were comparable between the two treatments. Conclusions: These findings underscore the efficacy of both PRGF and BXG in periodontal regeneration, with treatment decisions guided by patient-specific factors and clinical goals.

## 1. Introduction

Periodontal disease is a widespread oral health concern, affecting millions of individuals worldwide and posing a significant challenge to dental and periodontal care [1]. It is characterized by chronic inflammation of the periodontal tissues, leading to the degradation of the alveolar bone that supports the teeth and ultimately resulting in the formation of intra-bony defects [2,3,4]. These defects, representing localized pockets and bone loss, are pivotal in the progression of periodontal disease as they compromise the structural integrity of the periodontium and are associated with tooth mobility and tooth loss [5,6,7]. Addressing intra-bony defects is, therefore, imperative for the restoration of periodontal health and function.

Periodontal healing involves restoring the health of the tissues surrounding teeth after therapy, which can result in either repair or regeneration [8]. Repair is the re-establishment of tissue continuity without fully restoring the original architecture, often resulting in a long junctional epithelium or scar tissue. In contrast, regeneration seeks to completely rebuild lost structures, leading to new bone, cementum, and periodontal ligament formation, thus restoring both the structure and function of the periodontium [9].

Periodontal regeneration refers to the complete restoration of the lost or damaged periodontal structures, mimicking the essential wound-healing processes [10]. While several surgical techniques have been employed to create the optimal environment for periodontal regeneration, open flap debridement (OFD) or access flap surgery has shown promise in adjunct to a variety of biomaterials [11,12,13,14]. Nonetheless, these conventional strategies have limitations in achieving complete periodontal regeneration [15].

True regeneration can only occur through activating specific periodontal ligament-derived cells within the remaining periodontium, capable of differentiating into fibroblasts, cementoblasts, and osteoblasts [16,17]. The presence of a scaffold, cellular lineage and, most importantly, signaling molecules is essential for any tissue regeneration [17]. The cellular lineage can be obtained from the existing periodontal structures, while blood clots and bone grafts offer scaffold support [18]. Nonetheless, a crucial component often missing in these wound-healing events is the presence of signaling molecules.

Various therapeutic modalities have been employed to manage these challenging intra-bony defects, among which periodontal regeneration techniques have gained substantial recognition for their potential to promote tissue healing and regeneration. Specific methods within the realm of true regeneration have emerged as promising candidates for the augmentation of hard tissue regeneration within intra-bony defects, like the use of barrier membranes [19,20,21], bone grafts [22,23], stem cells [24,25], growth factors, and platelet concentrates.

Platelet concentrates derive from the patient’s own whole blood and include different products like platelet-rich plasma (PRP), plasma rich in growth factors (PRGF), and platelet-rich fibrin (PRF). Such autologous products harness the regenerative potential of platelets, playing a pivotal role in the field of periodontal therapy [26] by facilitating the healing of intra-bony defects and other periodontal conditions. PRGF has drawn increasing attention in the last 25 years due to its regenerative potential, thanks to a concentrated and biologically active portion of the patient’s blood plasma. PRGF is rich in growth factors and cytokines, which are instrumental in various cellular processes, including hemostasis, tissue healing, and regeneration [27,28]. The application of PRGF in periodontal therapy offers a minimally invasive and autologous approach, potentially enhancing the body’s natural regenerative capabilities.

Bone grafts include autografts, allografts, xenografts, and alloplastic biomaterials, each with distinct advantages and considerations [29]. They serve as scaffolds, promoting new bone formation through osteoconduction and, in some cases, osteoinduction. Osteoinduction stimulates immature cells to become bone-forming cells, osteoconduction provides a scaffold for new bone growth, and osteogenesis is the direct formation of new bone by osteoblasts. Together, these processes facilitate effective bone healing and regeneration [29]. Xenogenic bone grafts consist of bone materials typically sourced from bovine or porcine origins [30,31]. The use of xenogenic bone grafts provides a clinically established and readily available alternative for enhancing periodontal tissue regeneration through osteoconduction.

Both PRGF and xenogenic bone grafts alone possess unique advantages in the context of regenerative periodontology, which raises the question of which intervention may provide superior clinical outcomes. Thus, the present clinical study aimed to address this fundamental question by directly comparing the efficacy of PRGF and xenogenic bone grafts in the treatment of periodontal intra-bony defects. Our research endeavors to elucidate the relative benefits, potential limitations, and clinical applicability of these two treatment modalities, contributing valuable insights to the optimization of regenerative approaches for patients suffering from periodontal disease. The null hypothesis of this study is that there is no significant difference in the effects of using PRGF and BXG on the periodontal parameters evaluated in this study.

## 2. Materials and Methods

The study was carried out at the out-patient department of Periodontics, Institute of Dental Sciences, Siksha ‘O’ Anusandhan University, India, between June 2020 and September 2022, as part of a PhD dissertation submitted to the University of Milan, Italy. The protocol was approved by the Institutional Review Board and the Ethics Council of Siksha ‘O’ Anusandhan University. The following selection criteria were employed for including patients:

Inclusion criteria:

1. Patients aged between 30 and 50 years;
2. Patients suffering from STAGE III periodontitis with grade A/B [32];
3. Patients presenting with 2- or 3-wall IBDs ≥ 3 mm deep measured from the alveolar crest to base of the defect;
4. Patients presenting with a probing depth (PD) ≥ 5 mm;
5. Patients who are systemically healthy and do not have any conditions that would contraindicate surgery.

Exclusion criteria:

1. Patients who underwent previous periodontal surgical treatment;
2. Patients presenting with interdental osseous craters;
3. Immuno-compromised patients;
4. Patients showing poor oral hygiene maintenance even after thorough scaling and root planing;
5. Women who are pregnant and lactating;
6. Patients on any antibiotic and/or steroid therapy within the last six months;
7. Patients presenting with teeth affected by peri-apical infection.

### 2.1. Study Design

This prospective, comparative clinical study was designed to compare PRGF with bovine xenogeneic graft (BXG) for surgical management of periodontal intra-osseous defects. Based on a pilot study carried out with similar groups on eight patients and the mean difference observed using relative attachment level (RAL) as the primary outcome; 16 per group, totaling 32 sites, was found to be the estimated sample size, with 80% as the statistical power and a significance level set at 5%.

### 2.2. Patient Characteristics

Fifty-two patients presenting with GRADE A/B and STAGE III periodontitis were initially enrolled for the present study. All patients underwent meticulous Phase I therapy. A re-evaluation was carried out after six weeks to ensure the patient’s fitness to undergo surgery. Out of 52 enrolled patients, six failed to attend the re-evaluation appointment, and an additional six were excluded because they did not meet the predefined inclusion criteria. Finally, forty patients were randomized to receive one of the two treatments.

### 2.3. Outcomes

The clinical, radiological, and patient-reported outcomes were assessed at baseline and at various time points. The primary outcome of this study is RAL, and all other outcomes assessed were regarded as secondary. The details of each outcome and their methods of assessment are described below.

#### 2.3.1. Clinical Outcomes

- Plaque index (PI)—[33];
- Gingival index (GI)—[34];
- Probing depth (PD), in mm;
- Relative attachment level (RAL), in mm;
- Gingival marginal level (GML), in mm.

#### 2.3.2. Radiological Outcomes

Intra-bony defect depth (IBDD) in mm; measured on radiographs. The measurement was made from the deepest point of the intra-osseous defect to the imaginary line joining the adjacent cementoenamel junctions.

Intra-oral periapical radiographs (IOPAs) were made using the long cone paralleling technique. Position-indicating film holders were used to ensure stability. Customized bite blocks made from the putty index of patients were created and stored to ensure consistent positioning of the IOPAs at each post-operative recall interval.

For standardized exposure of the radiographs, the exposure time was set at 0.8 s, with a voltage of 70 kV and a current of 8 mA. The paralleling technique was used to prevent image overlap in the tooth’s interproximal areas. All radiographs were digitized using an 800 dpi scanner (HP Scanjet 3c/I, Hewlett Packard, Palo Alto, CA, USA).

#### 2.3.3. Patient-Reported Outcome Measures (PROMs)

The pain level experienced in the first seven days post-operatively from Day 1 to Day 7 was recorded using a visual analog scale (VAS) ranging from 0 to 100, where 0 corresponds to no pain, and 100 corresponds to severe pain.

The bleeding and swelling at the treated sited were recorded from Day 1 to Day 7 by asking the patients to rate them from 0 to 5 (0—Never, 1—Rarely, 2—Occasionally, 3—Quite Often, 4—Very Often)

### 2.4. Study Follow-Up

The follow-up of the study was carried out for 6 months. All the clinical and radiological outcomes were assessed at baseline, 3, and 6 months. And the PROMs were recorded post surgery until 1 week.

### 2.5. Randomization and Blinding

Randomization was carried out using the coin toss method soon before starting the surgical phase. In this way, allocation concealment was ensured. The study was single-blinded, as the assessor (Si.P.) was masked at all time points. Neither the surgical operator nor the patient could be blinded due to the nature of the two treatments.

### 2.6. Surgical Procedure (Figure 1)

All the periodontal surgery procedures were carried out by an experienced periodontist (S.P.). The patients were given a pre-procedural mouth rinse of 0.2% Chlorhexidine Gluconate (Hexidine, ICPA pharma, Mumbai, India). The surgical sites were anesthetized locally by administering 2% lignocaine (Lignox, Warren pharma, India).

**Figure 1:**
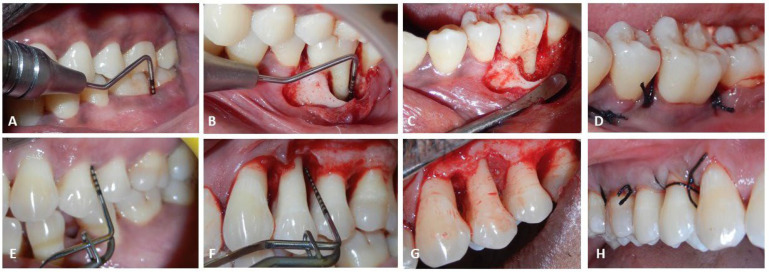
Surgical Procedure: (**A**–**D**) for BXG group; (**E**–**H**) for PRGF group.

Following adequate anesthesia, open access flap surgery was planned. A combination of crevicular and vertical incisions were placed to retract the flap and gain access to the defect. The defect was thoroughly degranulated using curettes (Standard Graceys. HuFriedy Group). The defect site was irrigated with saline and any remaining granulation tissues were completely removed. The defect site preparation was meticulously carried out to receive the active substitute.

### 2.7. For PRGF Group

#### 2.7.1. Preparation of PRGF

First, 9 mL of the patient’s blood was collected with 3.8% trisodium citrate as the anticoagulant. Once collected, the blood was subjected to centrifugation at 460× *g*. Centrifugation separates the blood into three distinct layers: the bottom fraction containing red blood cells, the middle fraction, also known as the “buffy coat”, containing white blood cells, and the upper fraction, plasma, is rich in platelets and growth factors.

The upper fraction was divided into two fractions: F1 (Fraction 1) and F2 (Fraction 2). F2 was isolated and transferred to sterile tubes, and was then activated using 10% calcium chloride, to form the PRGF gel.

#### 2.7.2. Placement of PRGF into Defect Site

The obtained PRGF gel was then placed into the intra-bony defect and condensed until the whole defect was filled.

### 2.8. For BXG Group

The infra-bony defects were packed and condensed up to an optimal level with the mixture of BXG (Bio-Oss, Geistlich Pharma, Switzerland) after proper debridement.

Single interrupted sutures using 6-0 monofilament suture (Ethicon) were placed to stabilize and secure the flap. The periodontal dressing was applied.

Patients were instructed with analgesics (paracetamol 500 mg) thrice a day for the next three days. Proper post-surgical instructions were provided to all patients, asking them to refrain from brushing at the operated area for the next seven days.

### 2.9. Statistical Analysis

SPSS software version 26.0 (IBM, New York, NY, USA) was used to analyze the data. The Kolmogorov–Smirnov test and the Shapiro–Wilk test were used to check the normality of the quantitative data distributions. Based on the normality, Student’s t-test was used to analyze the parametric data to compare outcomes between the groups, and the Mann–Whitney U test was used for the non-parametric set of data. A *p*-value of 0.05 was set as the level of significance.

## 3. Results

**Figure 2:**
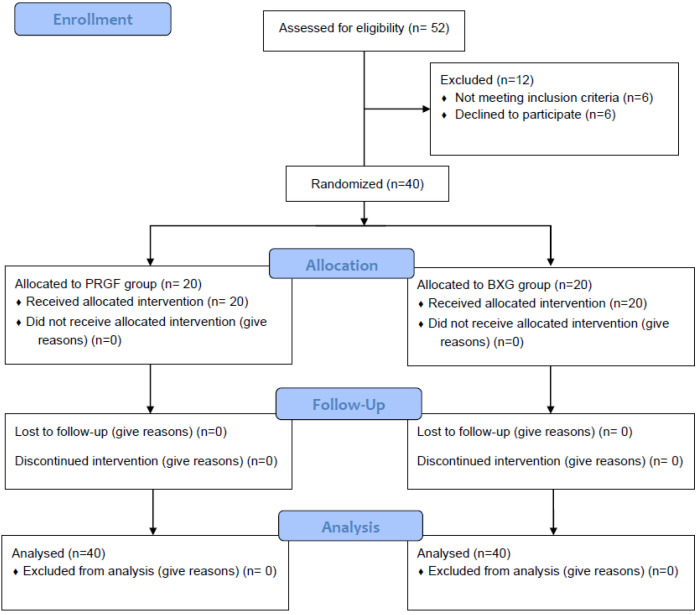
Flow diagram showing recruitment and follow-up of patients.

A total of 52 patients were assessed for eligibility, out of which 40 patients met the inclusion criteria and were randomly allocated to one of the two groups. No patients were lost to follow-up. A total of 20 patients in each group received the allocated intervention with either PRGF or BXG for management of intra-bony defects. (Figure 2). The demographic details of the patients are provided in Table 1.

### 3.1. Clinical Parameters

The PRGF group showed a significant improvement in GML at 3 and 6 months compared to the BXG group, indicating a potential advantage of PRGF in this aspect of periodontal health. However, other parameters such as PPD, RAL, PI, and GI did not show significant differences between the two groups (Table 2).

The results indicate that both the PRGF and BXG treatment groups experienced reductions in PPD over the study period. In general, the differences between the study groups were not statistically significant. However, the PRGF group exhibited statistically significant improvements in RAL and GML at the 3-month follow-up, indicating a potential advantage of PRGF in accelerating the improvement in the RAL and GML (Table 3).

**Figure 3:**
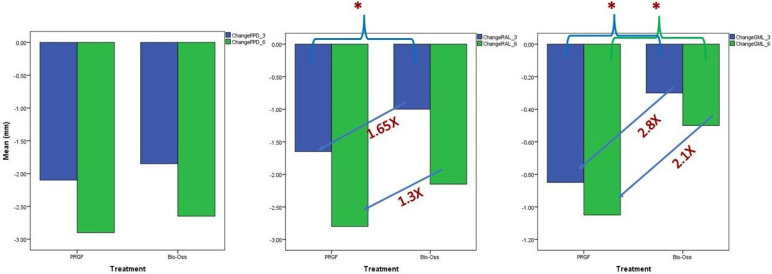
Change in PPD, RAL, and GML at the end of 3 and 6 months. * *p* < 0.05.

The reduction in the gingival recession was 2.8 times higher in the PRGF group compared to the BXG group at the end of 3 months and 2.1 times higher at the 6-month follow-up. Similarly, RAL gain was found to be 1.65 times greater in the PRGF group than the BXG group (Figure 3).

### 3.2. Radiological Parameters

The average reduction in the IBDD was 1.02 mm and 1.38 mm in the PRGF and BXG groups, respectively (*p* = 0.043) (Table 4).

### 3.3. Patient-Reported Outcomes (PROMs)

Both the PRGF and BXG groups experienced a reduction in pain over the post-treatment period, with pain levels decreasing as the days progressed. While there were no statistically significant differences in pain levels between the two groups on the first and second days, significant differences emerged on the third, fifth, and seventh days, favoring the PRGF group (Table 5).

The differences in post-operative bleeding between the PRGF and BXG groups were not statistically significant over the first week, except for a slight significance on the first day. Post-operative swelling also did not significantly differ between the two groups on any of the days post treatment.

## 4. Discussion

Periodontal regeneration in the context of intra-bony defects represents a crucial aspect of periodontal therapy, aiming to restore the lost periodontal tissues and prevent further disease progression [10,35,36]. In this clinical study, we explored the efficacy of two prominent regenerative approaches—plasma rich in growth factors and xenogenic bone graft—in the treatment of periodontal intra-bony defects.

The initial comparison of demographic and clinical characteristics between the PRGF and BXG groups showed a similar distribution of sex, age, smoking status, and the features of walled defects. This suggests that the randomization process was effective in creating comparable treatment groups, reducing potential sources of bias.

Our primary objective was to evaluate the regenerative potential of PRGF compared to BXG in treating periodontal intra-bony defects. The results indicated that both treatment modalities led to improvements in clinical parameters such as probing depth reduction and clinical attachment level gain. These findings align with the existing literature [37,38] that recognizes the regenerative capabilities of both autologous platelet concentrates and bone substitutes in periodontal therapy.

PRGF is derived from autologous blood and offers promising sources of an array of growth factors. PRGF contains essential growth factors, including PDGF-AB, TGF β1, and VEGF, capable of stimulating cell proliferation, matrix remodeling, and angiogenesis [39]. Research has demonstrated the positive effects of PRGF on bone regeneration, including its application in rabbit calvarial bone defects [40], tibial bone defects [41], peri-implant bone healing [42,43], periodontal pockets [44], and human extraction socket healing [45,46,47]. PRGF has also shown promise in treating human periodontal defects, including grade 2 furcation defects [48,49], which significantly improved attachment levels and reduced defect depth and extent.

Xenografts are derived from non-human sources, most commonly bovine or porcine origin, and have gained prominence in the field of periodontal regeneration. These graft materials are processed to eliminate potential immunogenicity and pathogenicity concerns, rendering them biocompatible and safe for clinical use. Xenografts offer a versatile solution for periodontal therapy, particularly in cases where autologous grafts may be limited, such as insufficient donor site availability [50].

Xenografts facilitate periodontal regeneration through several mechanisms, including osteoconduction and providing a scaffold for new bone formation. The graft materials serve as a matrix for bone-forming cells to adhere to, proliferate, and produce new bone tissue [51]. Moreover, some xenografts possess osteo-inductive properties, further stimulating the differentiation of progenitor cells into osteoblasts, thereby promoting bone formation within intra-bony defects [52]. Therefore, BXGs have established themselves as a valuable resource in periodontal regeneration, offering an effective solution for the management of intra-bony defects.

However, it is important to note that in the present study there were no statistically significant differences between the two treatment groups in terms of probing depth reduction and relative attachment level gain. This suggests that, within the study’s limitations and the specific patient population, PRGF and BXG have comparable efficacy in promoting periodontal regeneration in intra-bony defects. These results are in accordance with previous studies that have reported the effectiveness of both treatment modalities. 

The observed increase in gingival marginal level for the PRGF group compared to the BXG group at the end of the follow-up period is an interesting finding with potential clinical significance. The presence of creeping attachment in the PRGF group suggests that the treatment has not only been effective in filling intra-bony defects but has also had a positive influence on the reattachment of periodontal tissues, such as the connective tissue and gingival margin. This result suggests that PRGF may have a more favorable impact on the gingival margin’s position, which is an essential aspect of periodontal health and esthetics. The improvement in gingival marginal level in the PRGF group could be attributed to the regenerative potential of PRGF, which contains a concentrated and biologically active portion of the patient’s blood plasma, rich in growth factors and cytokines [39]. These components are known to stimulate tissue healing and regeneration, including the regeneration of periodontal tissues. However, it is crucial to interpret this result within the context of the entire study. The statistical significance, clinical relevance, and sustainability of this increase in gingival marginal level should be thoroughly analyzed.

While there were no statistically significant differences in pain levels between the two groups on the first and second days, significant differences emerged on the third, fifth, and seventh days, favoring the PRGF group. This suggests that the PRGF group reported lower pain levels after the second day of surgery. It is important to consider that, while there are statistically significant differences on certain days, the clinical significance of these differences may vary, and the overall patient experience should be taken into account. Pain perception can be influenced by various factors, including individual pain thresholds and the specific surgical techniques used. Therefore, these findings should be treated with caution.

The radiographic method was chosen to assess intra-bony defect depth because of its non-invasive nature and its ability to provide a comprehensive view of the bone structure. Radiographs are widely used in periodontal studies for visualizing bone loss and defect morphology, which are essential for evaluating treatment outcomes. However, it is important to recognize that the accuracy of this method can be affected by various factors, including the resolution of the imaging system, the angle of the radiographic projection, and the calibration of measurements. To minimize these shortcomings, standardization of the radiographic technique is crucial. This includes consistent imaging protocols, positioning of the patient, and calibration of measurements, which can help ensure more accurate and reliable assessments.

Both the PRGF and BXG group were found to be effective in reducing IBDD over the 6-month study period. It was found that the change in IBDD was significantly greater in the BXG group compared to the PRGF group. However, PRGF represents a unique approach because it consists of autologous plasma rich in growth factors, which stimulates tissue healing and regeneration directly from the patient’s own biological resources. This key distinction means that what we observe with PRGF is not merely a biomaterial filling, but rather a reflection of true tissue healing and regeneration. This insight underscores the importance of interpreting study results within the context of the treatment’s biological mechanisms and the potential for radiopacity to influence measurements when using biomaterials like BXG.

The outcomes of this study have several clinical implications. First, clinicians can confidently consider both PRGF and BXG as viable options, depending on patient preferences, clinical circumstances, and available resources. The lack of statistically significant differences between these treatments suggests that the choice may be based on individual patient factors and clinical considerations.

In the context of our study comparing the effectiveness of PRGF and BXG in periodontal regeneration for intra-bony defects, several potential confounding factors demand consideration. These variables, if left unaddressed, have the potential to influence the study outcomes. These confounding factors include patient compliance with post-treatment care and follow-up, variations in the baseline severity of periodontal disease, discrepancies in periodontal diagnosis and disease progression rates, concomitant medication usage, diverse oral hygiene practices, the presence of smokers in each treatment group, previous dental procedures undergone, socioeconomic disparities among participants, operator skill and experience, and differences in systemic health. Controlling for these confounding factors via our robust study design, strict inclusion and exclusion criteria, randomization, and appropriate statistical analysis is imperative to ensure the reliability and validity of our study’s conclusions regarding the relative efficacy of PRGF and BXG in achieving successful periodontal regeneration in intra-bony defects.

The comparable outcomes observed with PRGF and BXG may stem from their overlapping regenerative capabilities, as both aim to enhance bone healing, albeit through different mechanisms. PRGF promotes regeneration with growth factors that stimulate cell proliferation and osteoblast activity, while BXG acts as an osteoconductive scaffold for bone growth. In the two-wall and three-wall defects included in this study, both materials likely benefited from the natural regenerative potential of the pre-existing bony walls, which may have minimized differences. Also, variability in individual healing responses could have also played a role, further blurring potential differences. A longer follow-up and inclusion of diverse defect types might help clarify the long-term effectiveness of PRGF and BXG.

The limitations of this study may amount to a relatively small sample size and inclusion of a specific patient population, which may have influenced the generalizability of the results. Further research with larger and more diverse populations is warranted to strengthen the findings. Another limitation of this study is that the follow-up was limited to 6 months. While 6 months is generally sufficient for assessing periodontal regeneration and observing improvements in clinical parameters, longer follow-up periods could provide additional insights into the long-term stability and durability of the treatment outcomes over time, potentially revealing additional changes in bone density, clinical attachment, or probing depths that might not be evident in the shorter follow-up period.

## 5. Conclusions

In conclusion, both PRGF and BXG as substitutes were effective in promoting periodontal regeneration. Both treatment protocols proved equally effective with no statistical difference. However, PRGF exhibits a distinct advantage in enhancing gingival marginal levels in the short term and managing post-operative pain, as evidenced by early pain alleviation by the end of 3rd day post-operatively. These results suggest that clinicians can choose between PRGF and BXG based on patient-specific factors and clinical considerations, to tailor therapies to the individual needs of the patient. PRGF may be particularly advantageous for patients seeking faster soft tissue recovery. Future studies with larger sample size and more diverse population, and extended follow-up periods are warranted.

## Figures and Tables

**Table 1:** Demographic data of both groups.

		Treatment Groups		Total N	*p*-Value
		PRGF	BXG		
Sex	Female	12	12	24	
	Male	8	8	16	1.000 ^a^
Total		20	20	40	
Age (in Years)		52 ± 9	53 ± 10	40	0.679 ^b^
Smoker	N	15	11	26	0.185 ^a^
	Y	5	9	14	
Total		20	20	40	
Walled_defect	2	3	7	10	0.154 ^a^
	3	14	8	22	
	Combined	3	5	8	
Total		20	20	40	

PRGF = plasma rich in growth factors; BXG = bovine xenogeneic graft; ^a^ Chi-square test; ^b^ Student’s test.

**Table 2:** Clinical parameters at all time points.

Outcomes	Time Points	N	PRGF	BXG	*p*-Value
PPD (mm)	Baseline	20	6.55 ± 1.05	6.55 ± 1.19	1.000 ^a^
	3 months	20	4.45 ± 0.69	4.70 ± 0.86	0.398 ^b^
	6 months	20	3.65 ± 0.81	3.90 ± 0.91	0.602 ^b^
RAL (mm)	Baseline	20	12.60 ± 1.50	12.10 ± 1.25	0.260 ^a^
	3 months	20	10.95 ± 1.32	11.10 ± 1.45	0.718 ^b^
	6 months	20	9.80 ± 1.36	9.95 ± 1.28	0.721 ^a^
GML (mm)	Baseline	20	1.90 ± 0.79	1.90 ± 0.72	0.977 ^b^
	3 months	20	1.05 ± 0.69	1.60 ± 0.60	0.010 ^b^
	6 months	20	0.85 ± 0.75	1.40 ± 0.88	0.041 ^b^
PI	Baseline	20	0.80 ± 0.24	0.93 ± 0.23	0.046 ^b^
	3 months	20	0.62 ± 0.15	0.66 ± 0.15	0.414 ^b^
	6 months	20	0.54 ± 0.15	0.51 ± 0.16	0.565 ^a^
GI	Baseline	20	1.75 ± 0.44	6.55 ± 1.19	0.035 ^b^
	3 months	20	0.95 ± 0.22	0.80 ± 0.62	0.414 ^b^
	6 months	20	0.75 ± 0.44	0.65 ± 0.49	0.602 ^b^

^a^ Student’s *t* test ^b^ Mann–Whitney U test.

**Table 3:** Change at 3 and 6 months.

Outcomes	Change	N	PRGF	BXG	*p*-Value
PPD (mm)	Baseline-3 months	20	−2.10 ± 0.91	−1.85 ± 0.99	0.461 ^a^
	Baseline-6 months	20	−2.90 ± 1.37	−2.65 ± 0.99	0.738 ^a^
RAL (mm)	Baseline-3 months	20	−1.65 ± 0.59	−1.00 ± 0.79	0.007 ^a^
	Baseline-6 months	20	−2.80 ± 0.77	−2.15 ± 1.04	0.076 ^a^
GML (mm)	Baseline-3 months	20	−0.85 ± 0.49	−0.030 ± 0.57	0.009 ^a^
	Baseline-6 months	20	−1.05 ± 0.39	−0.50 ± 0.76	0.024 ^a^

^a^ Wilcoxon signed rank test.

**Table 4:** Intra-bony defect depth at baseline and 6 months in mm.

Treatment		IBDD-Baseline	IBDD 6 Months	Change at 6 Months
PRGF	N	20	20	20
	Mean	4.39	3.37	1.02
	SD	0.97	0.88	0.43
BXG	N	20	20	20
	Mean	4.46	3.08	1.38
	SD	0.70	0.69	0.62
*p*-value		0.183 ^a^	0.801 ^a^	0.261 ^a^

^a^ Student’s *t* test.

**Table 5:** Level of pain between both groups during week 1.

Treatment		Pain_D1	Pain_D2	Pain_D3	Pain_D4	Pain_D5	Pain_D6	Pain_D7
PRGF (20)	Mean	7.50	6.50	4.00	3.00	1.00	0.25	0.00
	SD	14.82	12.68	12.31	9.23	3.08	1.12	0.00
BXG (20)	Mean	22.00	20.00	16.50	12.50	8.00	7.00	5.25
	SD	29.31	25.55	29.25	26.33	18.24	15.59	10.94
*p*-value		0.183 ^a^	0.117 ^a^	0.036 ^a^	0.112 ^a^	0.177 ^a^	0.064 ^a^	0.019 ^a^

^a^ Mann–Whitney U test.

## Data Availability

The original contributions presented in the study are included in the article, further inquiries can be directed to the corresponding authors.
